# Nuclear localization of epidermal growth factor receptor (EGFR) in ameloblastomas

**DOI:** 10.18632/oncotarget.3919

**Published:** 2015-04-23

**Authors:** Núbia Braga Pereira, Ana Carolina de Melo do Carmo, Marina Gonçalves Diniz, Ricardo Santiago Gomez, Dawidson Assis Gomes, Carolina Cavalieri Gomes

**Affiliations:** ^1^ Department of Pathology, Biological Sciences Institute, Universidade Federal de Minas Gerais, Belo Horizonte, Brazil; ^2^ Department of Oral Surgery and Pathology, School of Dentistry, Universidade Federal de Minas Gerais, Belo Horizonte, Brazil; ^3^ Department of Biochemistry and Immunology, Biological Sciences Institute, Universidade Federal de Minas Gerais, Belo Horizonte, Brazil

**Keywords:** odontogenic tumors, cyclin D1, epidermal growth factor receptor, nuclear EGFR, therapy resistance

## Abstract

*Background:* Ameloblastoma is a locally invasive neoplasm often associated with morbidity and facial deformities, showing increased Epidermal Growth Factor Receptor (EGFR) expression. Inhibition of EGFR was suggested as a treatment option for a subset of ameloblastomas. However, there are resistance mechanisms that impair anti-EGFR therapies. One important resistance mechanism for EGFR-inhibition is the EGFR nuclear localization, which activates genes responsible for its mitogenic effects, such as Cyclin D1.

*Methods:* We assessed EGFR nuclear localization in encapsulated (unicystic, *n* = 3) and infiltrative (multicystic, *n* = 11) ameloblastomas and its colocalization with Cyclin D1 by using anti-EGFR and anti-lamin B1 double labeling immunofluorescence analyzed by confocal microscopy. Oral inflammatory fibrous hyperplasia and oral squamous cell carcinoma samples were used for comparison.

*Results:* Twelve cases of ameloblastoma exhibited nuclear EGFR colocalization with lamin B1. This positive staining was mainly observed in the ameloblast-like cells. The EGFR nuclear localization was also observed in control samples. In addition, nuclear EGFR colocalized with Cyclin D1 in ameloblastomas.

*Conclusions:* Nuclear EGFR occurs in ameloblastomas in association with Cyclin D1 expression, which is important in terms of tumor biology clarification and raises a concern about anti-EGFR treatment resistance in ameloblastomas.

## INTRODUCTION

Ameloblastomas are locally destructive aggressive benign neoplasms. Although they rarely metastasise, the surgical treatment of ameloblastomas usually results in facial deformities as well as in other morbidities. In this sense, molecular targeted therapy may be useful in aggressive and recurrent cases and *in vitro* experiments pointed to anti-EGFR therapy as an option for the treatment of a subset of *BRAF* wild-type ameloblastomas [1].

EGFR is a tyrosine kinase receptor involved in the transduction of extracellular mitogenic signals to different intracellular downstream signaling cascades. EGFR was identified as an important oncogenic factor in several cancer types [2]. Several studies demonstrated a strong EGFR expression in ameloblastomas [1, 3-7]. In primary ameloblastoma cells, treatment with EGFR monoclonal antibodies (cetuximab and panitumumab) or EGFR tyrosine kinase inhibitors (erlotinib, gefitinib and AG1478) suppressed cell growth, suggesting these therapies are effective for the treatment of a subset of ameloblastomas [1].

The treatment with EGFR-inhibitors is currently in clinical practice, having demonstrated important anti-tumor activity in patients with head and neck squamous cell carcinoma, metastatic colorectal cancer, lung cancer as well as breast cancer. However, major clinical response is only achieved in a small subset of patients, once response to these agents is limited by intrinsic and acquired resistance [8-11]. One EGFR-inhibition important resistance mechanism is the translocation of EGFR from the plasma membrane to the nucleus. This phenomenon has been described and associated with poor clinical outcome in breast cancer, oropharyngeal and head and neck squamous cell carcinoma and ovarian cancer [12-16]. Interestingly, nuclear EGFR localization is associated not only with resistance to the anti-EGFR therapies (cetuximab and gefitinib), but also with enhanced resistance to radiation and chemotherapy [17].

The function of nuclear EGFR has started to be clarified in 2001, when Lin and colleagues [18] demonstrated that the EGFR nuclear localization was found in highly proliferative tissues (such as uterus from pregnant mice, basal cells of normal oral mucosa and cancer cells from oral squamous cell carcinomas). In addition, they describe the role of nuclear EGFR as a transcription factor that can activate genes responsible for its mitogenic effects, such as controlling the expression of cyclin D1. We aimed to assess EGFR nuclear localization in Ameloblastomas and to investigate if it colocalizes with nuclear Cyclin D1.

## RESULTS

### Confocal immunofluorescence

Confocal microscopy was performed to observe the nuclear localization of the EGFR. Initially, we used the double labeling of EGFR with Lamin B1 in all ameblastomas. Lamin B1 was used to label the inner nuclear membrane since we wanted to evaluate EGFR intranuclear localization. Our results showed that 12 cases of ameloblastoma exhibited nuclear localization, including nine muticystic and three unicystic cases. This positive staining was observed mainly in the ameloblast-like cells. Three-dimensional reconstruction of serial confocal immunofluorescence images confirmed that the EGFR is found within the nucleus and also co-localizes with Lamin B1 (Figures 2 and 3). The EGFR nuclear localization was also observed in oral squamous cell carcinoma and in the epithelium of inflammatory fibrous hyperplasia.

**Figure 1:**
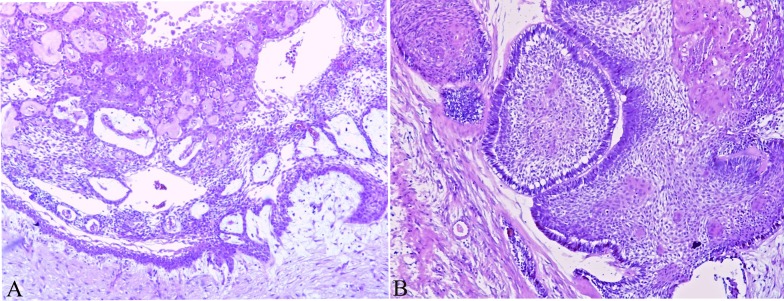
Histopathological picture of unicystic and multicystic ameloblastoma **A.** Unicystic ameloblastoma showing the presence of a connective tissue capsule lined by an ameloblastic epithelium, **B.** Multicystic ameloblastoma showing epithelial proliferation forming follicular structures lined by columnar cells that resembles ameloblasts. H&E staining; Original magnification 10x.

**Figure 2:**
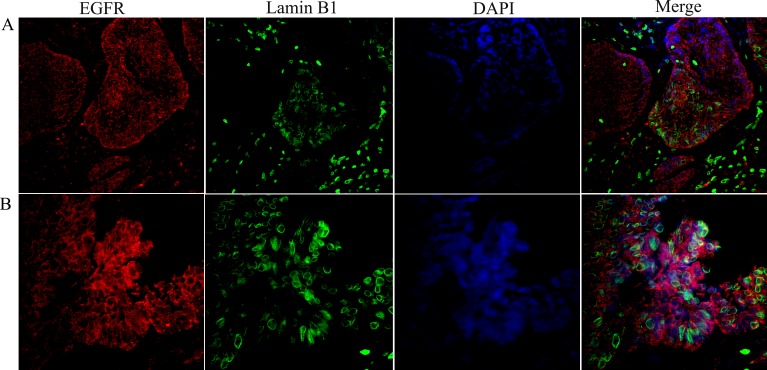
Co-localization of the nuclear EGFR with Lamin B1 in multicystic ameloblastoma Confocal microscopic analysis after immunofluorescence staining showing the nuclear localization of EGFR conjugated with Alexa Fluor 555 (red- left panels), Lamin B1 with Alexa Fluor 488 (green) and, nuclear staining with DAPI (blue). Merged images (right panels) demonstrate the co-localization of the nuclear EGFR with nuclei of ameloblast-like cells. Note that the EGFR is localized in both the cytoplasm and nucleus, and the nuclear staining presented in a punctiform manner within the nuclear envelope and chromatin (white arrows). Original magnification: **A.** 20x; **B.** 40x.

**Figure 3:**
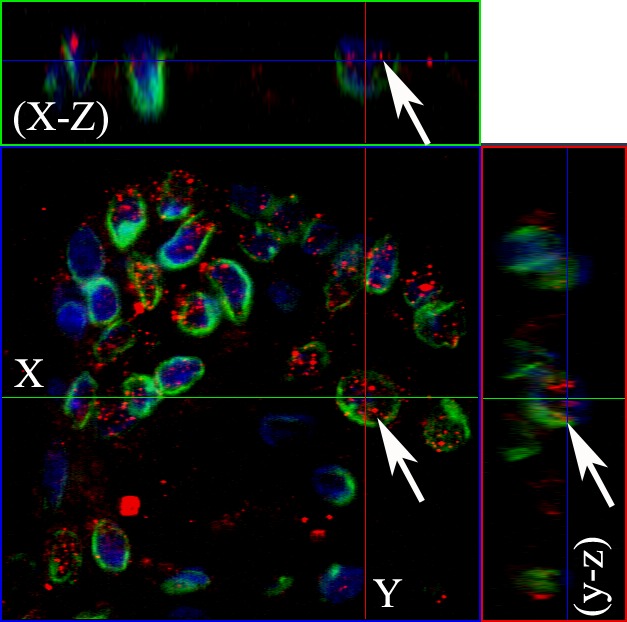
The nuclear EGFR co-localizes with Lamin B1 in ameloblastomas Serial optical sections were collected for three-dimensional reconstruction; x-z sections are shown at the top and y-z sections are shown on the right, to confirm nuclear co-localization of EGFR and Lamin B1. The EGFR and Lamin B1 antibodies were conjugated with secondary antibodies Alexa Fluor 555 (red) and Alexa Fluor 488 (green), respectively. Nuclear staining with DAPI is shown in blue. Original magnification: 63x.

**Figure 4:**
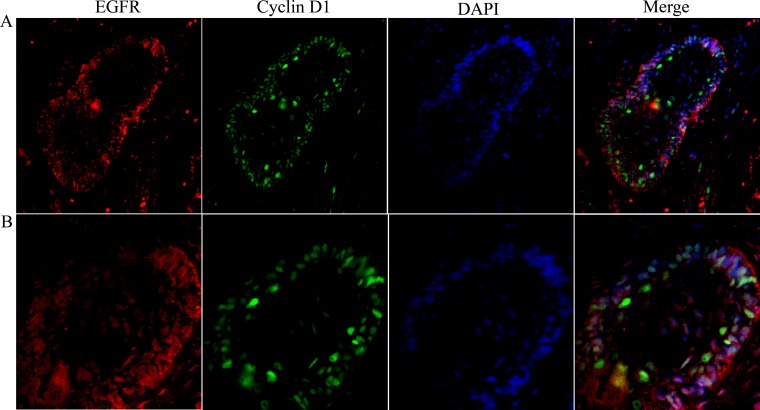
Co-localization of nuclear EGFR with Cyclin D1 in multicystic ameloblastoma Confocal immunofluorescent images showing the nuclear localization of EGFR co-localize with Cyclin D1 were obtained after staining Alexa fluor 555 (red- left panels), Lamin B1 and DAPI (green and blue respectively- middle panels) and Merge images (right panels) demonstrate that co-localization of the EGFR nuclear/Cyclin D1. Original magnification: **A.** 20x, and **B.** 40x.

**Figure 5:**
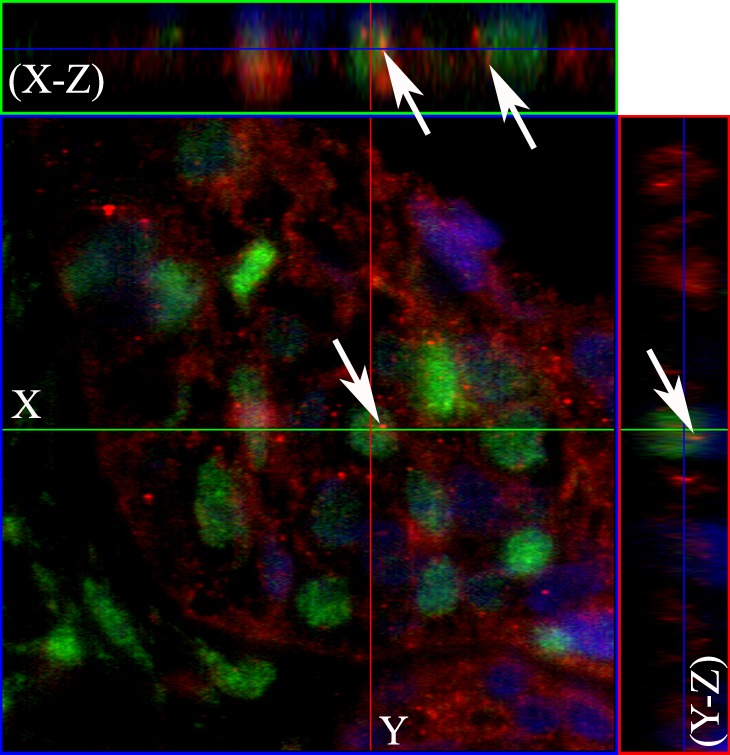
The nuclear EGFR co-localizes with Cyclin D1 positive cells The co-localization was observed using specific antibodies for the EGFR (red) and Cyclin D1 (green). Nuclear staining with DAPI is shown in blue. Serial optical sections were collected for three-dimensional reconstruction; x-z sections are shown at the top and y-z sections are shown on the right, to confirm nuclear co-localization of EGFR with Cyclin D1. Original magnification: 63x.

**Figure 6:**
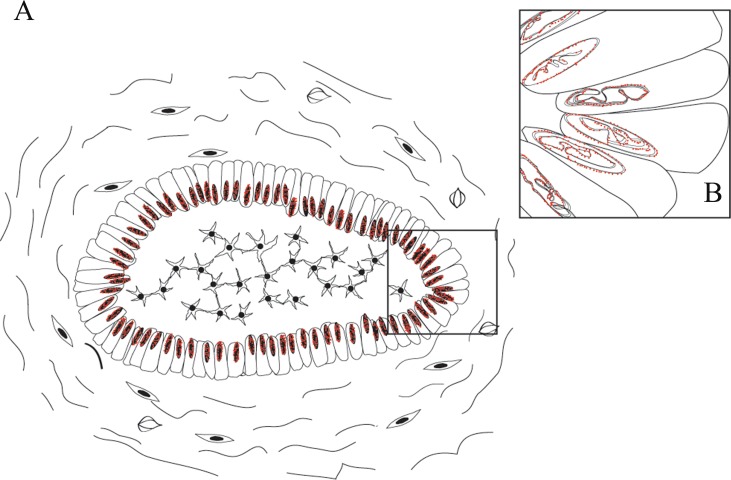
Schematic representations of nuclear EGFR localization in ameloblastomas **A.** EGFR nuclear localization was mainly observed in the ameloblast-like epithelial cells; **B.** Details of EGFR localization in the nuclear bilayer membrane. Two situations may occur, either the invagination of the inner nuclear membrane alone or invagination of both outer and inner nuclear membranes into the nucleoplasm [19].

Additionally, we evaluated if nuclear EGFR co-localizes with Cyclin D1 positive cells. We found that Cyclin D1 is co-localized with EGFR in unicystic and multicystic ameloblastomas, and the co-localization is clearer in the periphery of epithelial islands (ameloblast-like cells), which are cells that proliferate more (Figures 4 and 5). The location of the EGFR in the nucleus was schematically shown in Figure 6.

## DISCUSSION

The nuclear localization of EGFR has not been previously explored in ameloblastomas. We show that, similar to other tumor types, there is nuclear EGFR in both, unicystic and multicystic, ameloblastomas. We used the Lamin B antibody as a inner nuclear envelop marker, to prove that EGFR is accumulated along the inner nuclear membrane or with the chromatin. We found a punctiform distribution of EGFR within the nucleus. De Angelis and colleagues demonstrated by real time confocal imaging that few clusters of EGF/EGFR reach the nucleus after stimulation with EGF [20]. A recent published paper about EGFR signaling in immortalized ameloblastoma cell culture shows convincing images of EGFR nuclear localization, however, the authors do not mention this important feature [21].

Lin and colleagues [18] demonstrated experimentally that EGFR can bind to specific DNA sequences to activate gene expression, showing that Cyclin D1 is a potential target to nuclear EGFR. As Cyclin D1 is an important cell cycle regulator, they concluded that EGFR nuclear localization correlated with tissues with high proliferative activity. In line with these findings, our results point to a co-localization of nuclear EGFR and Cyclin D1. In addition, this phenomenon was more clearly observed in the peripheral ameloblast-like cells of the epithelium islands of follicular ameloblastomas, which are the cells known to have a higher proliferative activity. Similarly to our results, other studies in cancer samples revealed a correlation between nuclear EGFR localization and Cyclin D1 expression [22-23]. The confirmation of the direct binding of EGFR to the Cyclin D1 promoter in ameloblastomas can be further achieved by co-immunoprecipitation assay.

Our findings are important in terms of ameloblastoma tumor biology, elucidating this additional molecular feature of ameloblastoma pathogenesis. In addition, as in cell culture anti-EGFR treatment was proved effective for a subset of ameloblastomas (i.e. those *BRAF* wild-type) [1, 24], our findings bring to light a possible resistance mechanism to anti-EGFR therapy in such tumors, as shown for other neoplasms.

As a result of basic and translational research involving this nuclear EGFR localization and other resistance mechanisms against EGFR therapies, the development of new mechanism-based inhibitors, as well as combination therapies may be possible. [25]. In addition, the knowledge that nuclear EGFR is associated with resistance to different cancer therapeutics and correlates with worse survival in some tumor types motivate the seek for an alternative to target this nuclear receptor [17]. For other tumor types, such as ovarian cancer, the nuclear EGFR expression shows prognostic value [14, 23, 26]. We are describing the nuclear EGFR localization in ameloblastomas, however, future works with larger number of samples can reveal if this nuclear localization if of clinical significance.

In conclusion, we describe EGFR nuclear localization in unicystic and multicyst ameloblastomas, in association with Cyclin D1 expression.

## MATERIALS AND METHODS

### Tissue samples

Fourteen formalin-fixed and paraffin-embedded (FFPE) ameloblastoma samples were included in the study, comprising three unicystic (Figure 1A) and eleven multicystic cases (Figure 1B). Oral inflammatory fibrous hyperplasia and oral squamous cell carcinoma were used for comparison. The diagnoses were reviewed and confirmed in hematoxylin and eosin stained slides. The study was approved by the local ethics committee (protocol number 656.816).

### Confocal immunofluorescence

FFPE tissue sections (4 μm) were dewaxed, rehydrated and unmasked in trilogy solution (Cell Marque, Koclin, CA, USA) in a turbo convection steamer for 15 minutes according to manufacturer's instructions. Next, samples were rinsed in Phosphate Buffered Saline (PBS, 137 mM NaCl, 2.7 mM KCl and 10 mM phosphate buffer solution, pH 7.4) (Sigma-Aldrich, Carlsbad, CA, USA) solution, then incubated in PBS containing 0.2% Triton X-100 (Sigma-Aldrich) for 20 minutes and then blocked in PBS containing 1% bovine serum albumin (BSA, Sigma-Aldrich) for 30 minutes. The sections were then incubated with primary antibodies (either EGFR/Lamin B1 or EGFR/Cyclin D1) overnight at 4°C (Table 1) and rinsed 3 times for 10 minutes in PBS solution. Subsequently, sections were incubated with Alexa Fluor 488 and 555 (Life Technologies, Carlsbad, CA, USA) conjugated secondary antibodies diluted at 1:1000 for 1 hour at room temperature (Table 1), washed in PBS 3 times for 10 minutes and then mounted in prolong gold antifade reagent with DAPI (Life Technologies, USA). The negative control was included in all reactions, by omitting primary antibodies. Images were captured using a Zeiss 5 Live confocal (Carl Zeiss, Jena, Germany) microscope with a 10, 20, 40 and 63x objective lens. The samples were excited: (1) at 405 nm and observed at 420 - 460 nm to detect the nuclear staining with DAPI, (2) at 488 nm and observed at 505 - 550 nm to detect Alexa Fluor 488 and, (3) at 532 nm and observed with a LP560 filter to detect Alexa Fluor 555 staining.

**Table 1:** Detailed information on the primary and secondary antibodies

Primary antibody	Host	Clone	Dilution/Manufacturer	Secundary Antibody
Anti-EGFR	Mouse	Clone 8G6.2	1:200 Millipore	Alexa Fluor® 555 Goat Anti-Mouse IgG antibody
Anti-lamin B1	Rabbit	Polyclonal	1:1000 Abcam	Alexa Fluor® 488 Goat Anti-Rabbit IgG antibody
Anti-Cyclin D1	Rabbit	Clone SP4	1:100 Biogen	Alexa Fluor® 488 Goat Anti-Rabbit IgG antibody
